# Cross-talk between the HPA axis and addiction-related regions in stressful situations

**DOI:** 10.1016/j.heliyon.2023.e15525

**Published:** 2023-04-17

**Authors:** Marjan Nikbakhtzadeh, Hoda Ranjbar, Khadijeh Moradbeygi, Elham Zahedi, Mahnaz Bayat, Monavareh Soti, Mohammad Shabani

**Affiliations:** a Department of Physiology, School of Medicine, Tehran University of Medical Science, Tehran, Iran; b Neuroscience Research Center of Kerman, Institute of Neuropharmacology, Kerman University of Medical Science, Kerman, Iran; c Department of Nursing, Abadan Faculty of Medical Sciences, Abadan, Iran; d Clinical Neurology Research Centre, Shiraz University of Medical Sciences, Shiraz, Iran

**Keywords:** Addiction, Drug abuse, HPA axis, Stress, Neuro-inflammatory, Neurotrophic, Neurotransmitter

## Abstract

Addiction is a worldwide problem that has a negative impact on society by imposing significant costs on health care, public security, and the deactivation of the community economic cycle. Stress is an important risk factor in the development of addiction and relapse vulnerability. Here we review studies that have demonstrated the diverse roles of stress in addiction. Term searches were conducted manually in important reference journals as well as in the Google Scholar and PubMed databases, between 2010 and 2022. In each section of this narrative review, an effort has been made to use pertinent sources. First, we will provide an overview of changes in the Hypothalamus-Pituitary-Adrenal (HPA) axis component following stress, which impact reward-related regions including the ventral tegmental area (VTA) and nucleus accumbens (NAc). Then we will focus on internal factors altered by stress and their effects on drug addiction vulnerability. We conclude that alterations in neuro-inflammatory, neurotrophic, and neurotransmitter factors following stress pathways can impact related mechanisms on craving and relapse susceptibility.

## Highlights

• Stress is an important risk factor in the development of addiction and relapse vulnerability.
• There is a cross-talk between the HPA axis and addiction-related regions in stressful situations.
• Changing in the reward system, especially in response to stress, may result in drug-seeking behaviors.
• The reward system's neurotransmitters are altered by stress and addiction.
• Alterations in neurotransmitters following stress impact related mechanisms on craving.

## 1 Introduction

“Stress” is defined as the perception, evaluation, and response to harmful, threatening, or challenging events, and stressors can be either emotionally or physiologically based [1]. The stressful situation is inherent to human life, and the high level of stress is a major factor in starting the use of drugs that threaten the quality of life [2]. During stress, our body tries to regain its homeostasis and stability, which have become disrupted [3]. The hypothalamic-pituitary-adrenocortical (HPA) axis activates in response to stressors, causing glucocorticoids (GCs) (cortisol in humans, corticosterone (CORT) in rodents) to be released. Stress and GCs could elevate dopamine (DA) synthesis as a major neurotransmitter in the reward circuit [4]. As a result, the reward circuit, which is formed from neuronal connections including the prefrontal cortex (PFC), nucleus accumbens (NAc), ventral tegmental area (VTA), and amygdala, will be involved in drug reward reinstatement [5].

The effects of different types of stress, such as post-traumatic stress disorder (PTSD), pre-natal and post-natal stress, predator stress, and social deficit stress, on addiction have been evaluated in previous animal studies [6].

Furthermore, neuro-inflammatory and neurotrophic factors, as well as neurotransmitters, are also affected by addiction. In addition to being an indicator of internal stress, the HPA axis correlates with these factors that affect addiction. As a result, the goal of this study is to investigate the relationship between the HPA axis and addiction in order to determine how stress-induced changes in internal factors influence addiction vulnerability.

## 2 Rewarding behavior

Since reward motivates behavior and is necessary for survival, there is tremendous evolutionary pressure to remember the context of rewarding stimuli. This urge can be excessively strong, as in addiction, or weak, as in depression, which has anhedonia as a significant symptom (lack of pleasure in response to rewarding stimuli) [7]. Foraging and other behaviors that are motivated by rewards often involve a series of acts that start with a reward-seeking phase and end with an effort to retrieve and consume any rewards that were obtained during this activity [8].

## 3 Cores of reward circuit: the substrate for stress factors and drug abuse

The reward system origins in the medial forebrain bundle's anterior bed nuclei (glutamatergic), descends to the VTA (dopaminergic), and then ascends to the NAc (GABAergic) and PFC. Addictive drugs can directly or indirectly alter the reward system's function. In a bidirectional way, addiction may be caused by dysregulation of the reward system [9] by stress, which will be discussed below. In this review, our focus is more on the VTA and NAc areas.

## 4 The reward system's neurotransmitters are altered by stress and addiction

### 4.1 Dopamine

Stress-induced DA release in the NAc, which is facilitated by GCs. A number of mechanisms could account for the GCs' facilitation of DA release. 1) GCs increase DA synthesis through tyrosine hydroxylase (TH). 2) Monoamine-oxydase (MAO) is decreased by GCs. 3) GCs decrease DA reuptake. 4) GCs may also be able to influence extracellular DA via a number of neurotransmitters, including glutamate, opioids, GABA, and serotonin (5-HT). 5) GCs alter the dopaminergic neurons' rate of firing [10].

Several studies have shown that addictive drugs increase extracellular DA preferentially in the NAc in rats, non-human primates, and humans. Studies that have been done on self-administration (a type of operant training in which rats change their behavior and become addicts by receiving drugs as rewards) in local intracerebral areas have also confirmed that the NAc shell is the most sensitive location for DA-dependent rewards. Because of this, the NAc shell is the primary substrate for the dopaminergic terminal affected by acute exposure to addictive drugs in a naive individual. The bed nucleus of stria terminalis (BNST), which belongs to the extended amygdala and shares some anatomical similarities with the NAc, is also another sensitive area [11]. CRF (Corticotrophin-releasing factor)-R1 activation in the BNST is required for stress-induced cocaine-seeking relapse [12]. BNST sends CRF projections to the VTA that also co-release CRF. Glutamatergic and GABAergic projections from the BNST to the VTA have also been described [13].

Psychostimulants, by acting on the axonal terminal regions, increases DA release or inhibit its reuptake, leading to enhancement of the extracellular DA, while ethanol increase DA release by stimulating the firing activity of VTA DA neurons [14]. It has been suggested that tonic versus phasic DA releases mediate distinct aspects of addiction. Changes in burst firing may contribute to reward-specific information transmission, while changes in tonic firing are related to other aspects of addiction, such as withdrawal and craving. The excitatory effect of ethanol on VTA DA firing results in sustained increases in their tonic/burst firing, or both [15] (Table .1). While addictive drugs produce immediate increases in DA release during acute exposure, they also produce persistent changes in DA release during abstinence from the drug. Therefore, prolonged alterations in the firing activity of VTA DA neurons are noteworthy in this regard [16].

**Table 1:** Overview of neurotransmitters alteration following stress and addiction in the reward system.

		DA	GABA	Glu	AEA	NE	5-HT	Orexin
Chronic stress	VTA	↑	↓	↑	_	↓	↓	↓
	NAc	↑	↓	↑	↓	↑	↓	↑
Addiction	VTA	↑	↓	↑	↑	_	↑	↑
	NAc	↑	↑	↑	↑	↑	↑	↑

### 4.2 GABA

VTA GABA neurons, in general, provide local inhibition of VTA DA neurons. VTA GABA neurons, as a mediator of reward and aversion, are also involved in addiction, depression, and other stress-related disorders [17]. For instance, opioids hyperpolarized the VTA GABA neurons and disinhibited the VTA DA neurons [18].

CRF enhances the firing rate of dopaminergic and GABAergic neurons in the VTA [19]. Jennings et al. have illustrated that the footshock or a footshock-associated cue attenuated the firing rate of GABAergic neurons in the VTA, which transmit to the GABA neurons of the BNST. GABA afferents of BNST can inhibit the VTA GABAergic neurons and disinhibit the VTA DA neurons [20], while in an acute footshock stress situation, the BNST glutamatergic neurons showed an increment in firing rate, excited the VTA GABA neurons, and suppressed the dopaminergic firing rate [17]. As shown in acute stress, which increases the firing of VTA GABA neurons almost immediately, its lasting effects are more complicated because the plasticity of GABAergic synapses onto dopaminergic neurons is affected over a longer period of time. The GABAergic plasticity is thus lost over the following days, which removes the inhibitory effect of GABA on VTA DA firing [17].

Chronic unpredictable mild stress (CUMS)-induced depression results in a decrease in inhibitory synapse outputs, excitability, and excitatory synaptic reception in the NAc. Because the NAc contains GABAergic neurons, all of these attenuated changes in the subcellular compartments of GABAergic neurons result in the dysfunction of the NAc during chronic stress, which leads to major depression [21].

**Fig. 1:**
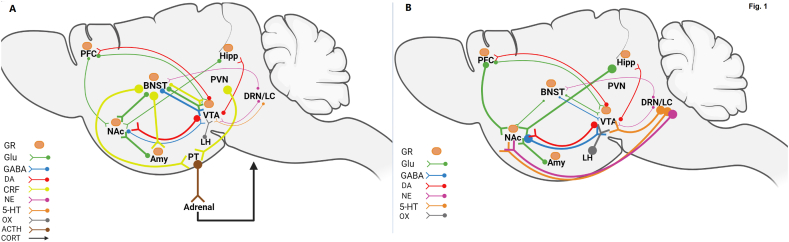
A schematic diagram of the brain reward circuit involved in addiction. The hippocampus, PFC, NAc, and amygdala receive projections from the VTA. In all reward and stress regions, GRs are present. A) As a result of the interaction between stress and the reward system, the LH-orexin neurons modulate the VTA DA neurons. VTA receives projections from BNST. PFC glutamatergic neurons communicate with VTA, DA, and NAc neurons. NAc, the hippocampus, and the BNST are modulated by glutamatergic neurons in the amygdala. NAc receives glutamatergic projections from the hippocampus. VTA and NAc receive serotonergic and norepinephrine projections from DRN and LC. B) The alteration of the reward system following addiction. The serotonergic pathways from DRN/LC to VTA and NAc are increased. The NE is also increased in the NAc. The GABA pathway from NAc to VTA was increased, as was the LH-oexin pathway to VTA. The thick arrow depicts hyperactivity, while the thin arrow depicts hypoactivity. Created with BioRender.com.

The basal level of extracellular GABA in the NAc increases 3 weeks after discontinuing treatment with cocaine injections [22]. Additionally, there is evidence for reciprocal presynaptic modulation between GABA, DA, and glutamate in the NAc, implicating altered GABA transmission in mediating the cocaine-induced changes to DA and glutamate transmission [23] (Table .1). Based on the above-mentioned studies, we can conclude that stress decreases the level of GABA in both the VTA and NAc, which are reversed by addiction (Fig. 1A and B). Therefore, drug-seeking behavior may also be influenced by changes in GABA levels, that needs further investigation.

### 4.3 Glutamate

Previous research has looked at other stress-induced changes in VTA DA neurons after drug abuse, such as hyperexcitability [24,25], upregulation of AMPAR subunit glutamate receptor 1 (GluR1), and NMDAR1 subunit. Acute swim stress increases the AMPA/NMDA ratio of excitatory synapses as much as drug abuse, which has a similar impact on VTA DA neurons [26,27], which requires GRs, glutamate A1 receptor (GluRA1), and NMDARs activation [26,27]. Western blot data showed social stress-induced cocaine cross-sensitization via augmentation of the VTA GluRA1 content [28]. Injections of CRF-receptor-2 (CRFR2) antagonists, but not CRFR1 antagonists, in the VTA area reduced glutamate and DA release and mitigated drug relapse [29].

The dopaminergic system is believed to mediate compulsive drug use at the level of the NAc; in contrast, the glutamatergic system's activity at the level of the NAc is mostly responsible for relapse after drug extinction [30]. Following stress, synaptic changes in the NAc shell reflect an enhancement of AMPAR-mediated currents but not of NMDAR miniature EPSCs. Stress-induced reinstatement of drug-seeking behavior in animals and relapse in humans may be related to altered processing of information via the plasticity of excitatory inputs [31]. Another study found that stress-induced glutamate-dependent DA enhancements in the NAc core which leads to the vulnerability to drug abuse [32]. Enhancement of glutamate release induced by conditioned cues and drug exposure in the NAc engages synaptic plasticity responsible for drug-seeking behavior [33] (Table .1). Overall, it is recognized that stress increases the expression of GluR1 and AMPA receptors in the VTA and NAc, which increases drug craving (Fig. 1).

## 5 The HPA axis and addiction

Stress is a physiological reaction to a physical or physiological threat [1]. In a stressful situation, stress signals from different regions such as the PFC, amygdala, and hippocampus are projected to the parvocellular neurons in the paraventricular nucleus (PVN) of the hypothalamus [34].

CRF stimulates adrenocorticotrophin (ACTH) hormone from the anterior pituitary, which subsequently triggers the secretion of cortisol/CORT from the adrenal glands. In chronic stress, the HPA axis is highly activated, and GCs released abnormally from the adrenal glands [35]. Cross-talk between the stress system and addiction at the three levels of the HPA axis has been reported in previous studies [36].

### 5.1 The pituitary gland, ACTH and addiction

The Pro-opiomelanocortin (POMC) system, ACTH, and β-endorphin modulate reward circuits in the brain and are involved in addictive behavior [37,38]. Smokers exposed to stress within four weeks of their relapse history had lower levels of ACTH, β-endorphin, and cortisol [31]. The β-endorphin level increased after alcohol consumption in the acute stage, but the negative feedback of cortisol on β-endorphine reduced the β-endorphine amount in the long term. ACTH is the product of β-endorphine cleavage. As a result, the reduction in ACTH was followed by a decrease in β-endorphins [38]. Chronic stress also increased the activity of the -melanocyte-stimulating hormone (-MSH) and melanocortin-4 receptor (MC4R), which resulted in a decrease in excitatory dopaminergic synapses on DA receptor type 1 (D1R)-expressing neurons in the NAc and the development of anhedonia [37].

### 5.2 The adrenal gland, glucocorticoids and addiction

At the third level, GCs play an important role in arousal, cognition, mood, immunity, and inflammatory reactions [39]. Moreover, studies have shown that there is a common pathway between self-administration of drugs and the release of GCs in an acute stressful situation [35]. Preclinical studies have revealed that the GCs antagonists administration in the basolateral amygdala (BLA) prevents stress-induced drug relapse [40]. Glococorticoid receptors (GRs) are expressed in the hippocampal formation, PFC, amygdala, and NAc, which are related to neural plasticity in drug abusers [[41], [42], [43]]. GRs in the NAc region play an important role in drug relapse in stressful situations by reducing DA clearance in this region [43]. In mice with knocked-out GRs genes, a reduction in firing of the VTA DA neuron and cocaine self-administration were observed [44].

The GRs of the hippocampus have a role in spatial memory formation that is induced by the Ca^2+^/calmodulin-dependent protein kinase II (CaMKII) and brain-derived neurotrophic factor (BDNF)-cAMP response element-binding protein (CREB) pathway [45]. Both drug abuse and GCs could have been inducing LTP in the ventral hippocampus, which relates to stress and emotional processing [46,47]. Additionally, Koenig and Olive demonstrated that GCs synthesis were blocked prior to ethanol consumption, which prevented further consumption [48]. It is clear from the aforementioned lines that stress is linked to increased HPA activity and GCs release. Therefore, GCs have a role in drug-related learning and memory [49] and also have a reciprocal relationship with drug susceptibility in drug consumers [50,51]. Below, we have discussed how certain types of drug addiction and the HPA axis are related.

## 6 HPA axis and alcohol addiction

It has been demonstrated that stress increases the probability of alcohol seeking in both healthy and addicted people [52]. Acute alcohol exposure whether supplied voluntarily or by an experimenter stimulated the production of CORT and ACTH, while prolonged treatment sufficient to cause dependency resulted in a dampened neuroendocrine state [53]. In people with severe alcohol use problems, protracted withdrawal and high levels of arousal can lead to HPA axis, GCs, and PFC dysfunction (AUDs). According to research, the HPA axis may be dysfunctional with binge/heavy drinking, and this is linked to non-dependent people's drive to drink [54]. Maternal separation stress (MS) mice showed a notable predilection for ethanol even at low dosages (0.1–1%), according to research on ethanol drinking behavior [55].

## 7 HPA axis and nicotin addiction

Stress makes it more difficult to resist the urge to use or seek out nicotine and intensifies the satisfaction that follows consumption [9]. Additionally, it has been discovered that human participants frequently attribute their prolonged nicotine abuse to stress [56]. During nicotine withdrawal, anxiety-like behavior and dysphoria (a negative and unpleasant affective state) are also connected to CRF [57]. Following long-term nicotine usage, abrupt withdrawal led to the HPA axis' hyperactivity [58].

## 8 HPA axis and opioid addiction

The group with maternal deprivation (MD) exhibited a greater level of CORT. In all of the brain regions, including the hippocampus and the NAc, MD rats' expression of the BDNF and GR genes was shown to have significantly decreased. Additionally, MD rats showed a substantial increase in the expression of the opioid receptor in all of the brain regions. According to previous findings, MD causes changes in the way the HPA axis works, the amount of BDNF in the body, and the opioid receptor system that make people more susceptible to morphine as adults [59]. Research on lab animals has linked the development of opiate self-administration behaviors and the progression to opiate dependency to the deregulation of numerous brain stress-responsive systems and the HPA axis [60,61]. Following the expression of conditioned place aversion (CPA), CORT levels rose. Prior to naloxone, pre-treatment with the selective CRFR1 antagonist CP-154,526 reduced the extinction duration and inhibited morphine-withdrawal-induced unpleasant memory consolidation [62].

## 9 VTA: stress and addiction

Dopaminergic neurons, which make up 65% of the VTA, are part of the reward prediction [17]. Recent studies revealed that CRFR1 and CRFR2 are important in the VTA response to social stress [63], and later cocaine “binge” self-administration was facilitated by these receptors’ existence [64]. Intermittent stressors have a lot of effects on different dimensions of substance consumption, which are mediated via VTA DA system activation, like psychomotor stimulant sensitization, conditioned place preference (CPP) enhancement, augmentation of cocaine self-administration, amphetamine, heroin, and cocaine seeking relapse [65].

The activity of VTA DA neurons participates in cognition, motivation, and locomotor activity, and it is widely implicated in reward-seeking such as drug abuse, and brain self-stimulation [66]. Morphine and ethanol have a reinforcing effect on VTA DA neurons by activating μ-opioid receptors [67,68]. It has been shown that VTA DA neuronal activity decreases in withdrawn rats after chronic morphine exposure [69] or ethanol administration [70].

## 10 NAc: stress and addiction

VTA sends the DA projections to NAc [71], which is another critical region of the reward circuit. The NAc as a major part of the ventral striatum is composed of GABAergic medium spiny neurons (MSNs) [72] which play an important role in responding to rewarding or aversive stimuli [73]. MSNs in the medial NAc shell inhibit the medial VTA DA neuron, whereas the lateral NAc shell neurons inhibit the VTA GABAergic interneurons and disinhibit the DA neurons. Hence, in a reverse direction, the DA neurons return to lateral NAc shell neurons and activate D1Rs [74]. D1R-MSNs in NAc convey reward signals, while D2R-MSNs encode aversion [75]. Drug consumption for a long time or exposure to chronic social defeat stress play an important role in the plasticity of MSN neurons in NAc, which is mediated via upregulating the D1Rs on MSN neurons and downregulating the D2Rs of these neurons in resilient animals [76]. Changes in NAc caused by stress can stimulate drug-seeking behavior as well as synaptic changes in the level of VTA projection and the lateral NAc shell [77]. Prenatal stress increased D2Rs expression in adult offspring's NAc; however, D2Rs were reduced in rats given nicotine, indicating that stress increased vulnerability to nicotine addiction [78]. In addition, restraint stress increased the response to drugs by increasing DA release in the NAc core [32]. Collectively, changing the reward system, especially in response to stress, may result in drug-seeking behaviors (Fig. 1).

## 11 The indirect effect of other neurotransmitters system following stress leading to addiction vulnerability

### 11.1 Endocannabinoid system

The endocannabinoid system (ECS) as a neuromodulatory system has a role in CNS development, synaptic plasticity, and the response to endogenous and exogenous threatening factors [79]. ECS presence in corticolimbic structures like the PFC, amygdala, and hippocampus has an important role in stress and anxiety-like behavior in adults and exerts this effect by regulating the HPA axis [80]. In a normal state, the HPA axis activity is constrained by the basal level of anandamide (AEA) in the BLA, but during stressful situations, CRF signaling coordinates a breakdown of tonic AEA activity to encourage a state of anxiety. When the effect of ECS has vanished, the HPA axis escapes constraint and GCs concentration increases [81]. AEA is an endogenous ECS that is synthesized and released by postsynaptic terminals, activates cannabinoid receptor type 1 (CB1Rs) in presynaptic terminals, and suppresses presynaptic neurotransmitter release [82].

Stress has different effects on the ECS depending on the brain region and duration of exposure. Both acute and chronic stress generally cause a reduction in the content of AEA in the brain tissue [83]. Maternal separation disrupts emotional memory formation and increases cannabinoid sensitivity by altering CB1R signaling [84]. CUMS could induce ECS signaling deficits in the NAc by impairing CB1Rs function [85]. Dysregulated neural plasticity, increased stress reactivity, unpleasant affective states, and cravings that fuel addiction are all caused by impaired ECS signaling [86]. Stress-induced alcohol consumption is influenced by ECS signaling, but not stress-induced relapse after quitting. Similar inconsistencies with cocaine have been discovered, where CB1R antagonistism does not prevent footshock-induced resumption of cocaine-seeking behavior under stress [87], but it has been discovered to stop the resumption of cocaine-seeking behavior brought on by swim stress [88] or by administering CRF directly [87]. Additionally, CB1R activation speeds up the VTA DA neurons' rate of firing, which thus increases drug seeking and reinforces marijuana's addictive properties [89]. Since ECS signaling was previously mentioned, it is plausible that the stress modality itself could have varied impacts on this situation's ECS signaling, with ECS signaling potentially being critical for some forms of stress-induced cocaine reinstatement.

Nicotine self-administration reduced baseline VTA dialysate oleoyl ethanolamide (OEA) levels and increased AEA release during nicotine intake [90]. Chronic ethanol exposure and traumatic stress have an impact on cannabinoid components in limbic regions [91]. It is likely that CB1R antagonists have antagonizing effects on alcohol and nicotine reward due to a diminished ability to increase NAc DA release. Blockade of CB1Rs, specifically in the VTA and NAc, reduces alcohol consumption [86] (Table .1).

### 11.2 Norepinephrine

Locus coeruleus (LC) is the region containing the norepinephrine (NE) neurons. In confronting the stressor, LC receives the projections from the paragigantocellularis nucleus of the brainstem, which have previously been enriched via CRF fibers from PVN, and BNST which led to the anxiety-like behavior [[92], [93], [94]]. BNST also receives projections from NE neurons of the LC [95]. Multiple kinds of adrenergic receptors are expressed on VTA DA neurons, which might mediate the interaction between VTA DA neurons and LC-NE inputs. Activation of VTA α1 and β3 adrenergic receptors reversed social avoidance behavior in previously identified susceptible mice and normalized the pathological hyperactivity of VTA→NAc DA neurons. Based on this phenomenon, it appears that the LC-NE system and the VTA→NAc reward circuit might be synaptically relayed by the adrenergic receptors α1 and β3 [96]. It was discovered that resilient mice released more NE from their LC neurons that project towards the VTA, suggesting that NE plays a role in mood regulation in the VTA [97]. Findings revealed that α2 adrenergic receptor agonists prevent stress-induced craving in human cocaine-dependent subjects. Additionally, it has been shown that β2 adrenergic receptor activation of a CRF-releasing projection from the BNST to the VTA is necessary for stress-induced cocaine use. In this regard, the findings show that β adrenergic receptor blockade in the BNST prevents footshock-induced reinstatement in rats [98].

Social isolation is associated with an increased response of DA and NE in the NAc and increased sensitivity to ethanol-mediated stimulation of NAc DA and NE release [99]. However, chronic stress can also attenuate the LC firing rate [100] (Table .1). Chronic nicotine self-administration lowered PVN NE release produced by footshock stress in rats, which could modify CRF neurons in this region and lead to stress hypersensitivity during chronic nicotine use [101]. Acute nicotine increased stress-induced increases in plasma corticosterone and epinephrine, implying that the stress-relieving effects of cigarette smoking are not mediated by a decrease in peripheral sympathetic nervous system activation [102]. CRFR1 and α2 noradrenergic receptor may have a role in the heightened anxiety-like behavior observed following acute heroin withdrawal in rats, potentially through increasing the release of NE in stress-related brain areas [103]. Psychological stress may increase cocaine-seeking behavior in chronically addicted individuals or animals, and medications that reduce NE transmission may prevent stress-induced reinstatement of usage [104]. As a result, NE alters during chronic stress, intensifying the effects of stress and contributing to increased craving (Fig. 1).

### 11.3 Serotonin

Serotonin (5-HT), which is released by the dorsal raphe nucleus (DRN), has an important role in psychiatric disorders [105]. The previous consumption of psychostimulants or opioids accompanied by chemical or physical stressors elevates the sensitivity of 5-HT neurons in DRN by increasing the GABA_A_ receptors on these neurons [106]. In stressful circumstances, the CRF influences the DRN-5-HT system by modulating the serotonergic and GABAergic neuron dendrites [107]. Because the CRF impression on GABAergic neurons is more frequent, the electrophysiological studies indicated that the 5-HT neurons are inhibited indirectly by CRF and CRFR1 activity [105,106]. There is a complex regulation of DA release within reward circuitry that can be attributed to the multiplication of 5-HT receptors and their location on different neurons. 5-HT receptor subtypes exert excitatory effects on DA release in the NAc as well as discharge activity in VTA DA neurons that project to the NAc in substance abuse [105]. Serotonergic hypofunction, which is associated with dysphoric mood, can alter the susceptibility to stress-induced drug reinstatement [108]. Similarly, the CRF of the amygdala, by decreasing the 5-HT release in addicted rats, may be involved in aversion that is accompanied by drug withdrawal [105]. Stress reduces the reward responses of DRN 5-HT neurons and VTA DA neurons, resulting in anhedonia, a key symptom of depression. The negative effect of stress on reward responses suggests that some reduction in the reward responses of both 5-HT and dopaminergic neurons might underlie stress-induced anhedonia [109]. By decreasing 5-HT1B receptor stimulation, stress decreases 5-HT tone in the NAc of male mice to promote aversion and potentiate cocaine preference [110]. CRFR1 activation reduces 5-HT release and activates coping behavior in rats exposed to acute swimming stress [105]. However, when exposed to repeated swim stress, CRF aggregation impresses on CRFR2 and excites the 5-HT neurons, revealing passive coping behaviors such as avoidance, denial, self-blame, and immobility [105,111] (Table .1). As a result, low levels of 5-HT contribute to substance abuse initiation and relapse in acute stress conditions, whereas CRF increases 5-HT release from DRN neurons in chronic stress conditions [105]. Because 5-HT levels in reward circuits decrease with stress and increase with addiction, one reason for the proclivity to become addicted could be to compensate for the decrease in 5-HT (Fig. 1).

### 11.4 Orexin

Orexin (OX) neurons in the lateral hypothalamus (LH) secrete two neuropeptides, including OXA and OXB [112]. Evidence suggests that OXA signaling is more involved in reward seeking, whereas OXB signaling is associated with arousal and stress responses [[113], [114], [115]]. In some cases, such as chronic stress, compulsive drug craving, and chronic relapse, orexin neurons send projections to the hypothalamic stress systems, which activate CRF-containing neurons [112,116,117]. CRF stimulates orexin release in the LH, which is a factor involved in the activation and maintenance of arousal that is associated with the stress response and activates in response to cocaine, morphine, and their cues [118,119]. The LH is very important in motivating cocaine consumption by acting on the orexin factor [120]. Hyperarousal and withdrawal physical reactions observed after drug consumption increase orexin expression in LH neurons [121]. CRF terminals make direct synaptic contacts with orexin neurons, which express the CRF-R1/2. CRF and orexin have well-defined roles in drug-seeking behavior by regulating PFC-mediated addiction-like behaviors [122]. The induction of a stress-like state by orexin results in the reinstatement of cocaine seeking [123]. OXA projections into the paraventricular thalamus (PVT) induce cocaine-seeking behavior, which is perhaps promoted by CRF function in mediating stress and anxiety-like responses, which are well-known factors implicated in cocaine seeking in abstinent individuals [124]. Furthermore, the OXA antagonist reduces cocaine self-administration and stress-induced relapse [125].

The role of orexins in reward is partly explained by activating dopaminergic neurons in the VTA. Additionally, intra-VTA injections of orexin increase the levels of extracellular DA in NAc [126]. Cocaine exposure potentiates the synaptic plasticity via OXAR1 on VTA DA neurons and increases the firing rate of these neurons [127]. Orexin neurons project abundantly to the VTA and NAc and are activated by cues indicating food and drug rewards. OXA injections induce drug-seeking behaviors, suggesting that they are also involved in the reinstatement of reward-seeking behaviors [126]. In the NAc, chronic drug exposure has been found to result in long-term up-regulation of OXBR [115] (Table .1).

Based on previous studies, chronic stress often reduces the number of orexin-induced action potentials in DRN-5-HT neurons [128]. Stress reduces orexin, which, in turn, reduces 5-HT activation and leads to stress-induced drug craving. Chronic stress also leads to decreased prepro-orexin mRNA levels, reducing the number of orexin neurons in the VTA [129]. Orexin neurons also send many projections to the DRN [130]. However, recently, it has been shown that, following the stress condition, the neuropeptide S (NPS) of the brainstem also activates the LH orexinergic neurons [131]. Therefore, previous findings show that stress alters orexin secretion, which can mediate drug craving (Fig. 1).

### 11.5 The BDNF

BDNF is distributed in the cerebral cortex, hippocampus, amygdala, and hypothalamus [132], and it is contributing to neurogenesis and synaptic formation, neuronal differentiation, migration, and survival [133]. Early life separation stress increased the CRF and tyrosine hydroxylase (TH) in the hippocampus, NAc, and PVN, resulting in a reduction in the BDNF content in these regions in the offspring of mice [134]. By regulating the HPA axis, BDNF has an anxiety-like effect and is involved in neuroendocrine homeostasis [135].

There has been considerable interest in the effect of BDNF on reward-related neuronal circuitry. BDNF has been implicated in mediating synaptic plasticity associated with cocaine abuse as well as cocaine-induced behaviors, namely CPP, behavioral sensitization, and cocaine self-administration [136]. Cocaine exposure increases BDNF gene expression in the VTA, which has been linked to increased drug seeking [137].

Following intermittent social defeat stress and opiate consumption, μ-opioid receptors expression increased BDNF expression [138,139]. Episodic social defeat in rats with a cocaine abuse background increases the BDNF content of the VTA region [140,141]. While in continuously stressed rats, DA and BDNF content showed a reduction in the VTA that has a suppressive effect on cocaine seeking [142]. These conflicting findings may depend on the time of drug consumption, as studies showed that in the VTA, BDNF was significantly elevated after 10–15 days of withdrawal but not on withdrawal day 1 [143]. In the PFC, significant increases in BDNF expression were observed on withdrawal days 8 and 14, but not on withdrawal days 1 or 3 [144]. Therefore, cocaine administration regulates BDNF levels in a complex manner that varies depending on the addiction phase (e.g., acquisition/maintenance; early/late withdrawal).

It has been reported that the maternal separation, via decreased BDNF exon IV levels in the PFC area, leads to the increased exposure of adolescent mice to cocaine [145]. These mice showed a susceptibility to alcohol consumption in stressful situations in adulthood [134]. Chronic ethanol treatment reduced BDNF mRNA levels in mPFC, while forced swim stress (FSS) increased ethanol consumption [146]. Chronic stress inhibits BDNF signaling in the PFC, which is activated by cocaine and plays an important role in the development of cocaine side effects [147]. As a result, chronic stress and long-term drug consumption reduce BDNF, while BDNF increases following acute stress. Previous studies found that cocaine self-administration in rats induced a transient increase in BDNF in the NAc. Endogenous BDNF enhancement in the NAc subsequently reduced cocaine self-administration and attenuated relapse [148]. Thus, following stress or drug abuse, BDNF may have local-dependent alterations that are necessary to mediate or prevent cocaine-seeking behavior.

### 11.6 The neuro-inflammatory factors

There is a lot of evidence that chronic stress is linked to inflammatory processes, especially those that have to do with how the inflammatory cascade is controlled [149]. Stress and GCs sensitize the innate immune responses to proinflammatory cytokines [150]. Furthermore, stress can activate microglia and exert its function as an enhancer of microglial and glial mediators, contributing to the development of drug abuse [151,152]. Glial mediators include tumor necrosis factor-alpha (TNF-), interleukin 1 beta (IL-1), interleukin 6 (IL-6) and chemokines [153]. IL-1 and IL-6 stimulate the HPA axis and raise ACTH and CORT levels [154,155]. TNFα release from striatal microglia has been able to downregulate the AMPAR subunits in the striatum, a critical region for the motor and reward systems that receive glutamatergic and dopaminergic inputs [156]. It has been reported that the expression of the anti-inflammatory cytokine IL-10 in the NAc area reduces morphine-induced glial activation and prevents morphine CPP relapse in adulthood [157]. Furthermore, Ibudilast (an anti-inflammatory drug) used as a glial pro-inflammatory or TLR4 (inflammatory cytokine production initiator) antagonist reduced morphine-induced pro-inflammatory cytokine, morphine withdrawal behavior, and morphine-induced DA efflux in the NAc [158,159]. From the above lines of evidence, it can be concluded that anti-inflammatory or antagonistic inflammatory factors during stress can reduce the desire for drug abuse by affecting the reward pathway (Table .2).

**Table 2:** The reciprocal effect of HPA axis and internal factors.

Internal factors	Type of stress/drugs	The reciprocal effect of HPA axis and internal factors	Effect on addiction	Ref
Cannabinoids	Maternal separation in mice CUMS in mice Prolong alcohol and nicotine exposure Intermittent footshock stress, CRF, and cocaine in rats Swim stress and cocaine administration in mice CUMS and alcohol consumption in mice Single-prolonged stress and chronic intermittent ethanol in mice	The HPA axis increases sensitivity to cannabinoids Stress deficits in ECS signaling in the NAc CBR1 signaling dysfunction: increased stress responsivity Blocking of CB1R decreased the craving behavior induced by CRF Blocked by CB1R decreased the stress-induced cocaine-seeking behavior Alcohol reduced CB2R gene expression reduction, and CB2R agonist increased the alcohol consumption in stressful mice In stressful mice that received alcohol, AEA content was diminished	– – Increased the alcohol and nicotine consumption Increased the cocaine craving in acquisition phase not the reinstatement Increased the stress-seeking behavior for cocaine relapse Increased alcohol consumption –	[84] [85] [87] [88] [160] [91]
Norepinephrine	Stressful situation in human with cocaine consumption Footshock stress, cocaine consumption in rats Social isolation Footshock stress, nicotine abusing in rats Restraint stress, heroin abusing in rats Psychological stress, cocaine abusing	α2 receptor agonists prevented stress-induced craving in subjects β2 receptor activated the CRF-releasing from the BNST to the VTA Stress increased sensitivity to ethanol which is mediated the stimulation of NAc DA and NE release Chronic nicotine self-administration: attenuated the PVN norepinephrine release CRFR1 and α2 receptors caused the anxiety-like behavior during acute heroin withdrawal Stress increased the cocaine-craving in chronic abuser individuals or animals, and norepinephrine antagonist reversed the stress-induced relapse	Decreased the cocaine craving Increased the cocaine craving Increased the alcohol craving – – Increased the relapse	[98] [161] [99] [162] [103] [104]
Serotonin	Stress related to psychiatric disorders in rats Drug abuse in rats Forced swim stress, cocaine consumption in mice	The 5-HT neurons are inhibited indirectly by CRF and CRFR1 activity. 5-HT showed the excitatory effects on DA release in the NAc in substance abuse. The CRF decreased the 5-HT release Stress decreased the 5-HT tone in the NAc and promoted the aversion of cocaine preference	– Increased the drug abusing Decreased the drug abusing	[106,107] [105] [163]
Orexin	Stress and cocaine	OXA induced cocaine-seeking behavior	Increased the cocaine self-administration and stress-induced relapse	[124]
BDNF	Early life separation in mice offspring Intermittent social defeat stress in rats Episodic social defeated in rats Maternal separation in mice Chronic ethanol treatment and forced swim stress in mice	Stress reduced the BDNF content in the hippocampus, NAc, and PVN Stress up regulated the BDNF expression in the VTA Stress with a cocaine abuse background, increase the BDNF content of VTA Stress decreased BDNF in the PFC Ethanol and stress: reduced in BDNF mRNA levels of mPFC	Susceptibility to ethanol consumption and reinforcement Increased the stress-induced substance abuse Increased the drug sensitivity Increased the cocaine sensitization Increased the ethanol consumption	[134] [138,139] [140,142] [145] [146]
Neuroinflammatory	Morphine administration in rats	Ibudilast * and TLR4 ** mitigated the morphine-induced DA in the NAc	Reduced the morphine abuse	[158,159]

*An anti-inflammatory drug, ** TLR4: Toll-like receptor 4 (Inflammatory cytokine production initiator).

## 12 Conclusion

There is considerable evidence from preclinical and clinical studies that indicates an association between highly stressful situations and addiction vulnerability [164,165]. Stressor exposure causes long-lasting neuroendocrine and physiological changes in brain regions involved in learning, motivation, and stress-related adaptive behaviors, which are briefly discussed below as concluding remarks of points. 1) Stress increases the HPA's activity and GCs release. It also enhances drug-related learning and memory and increases the susceptibility to drug use. 2) VTA DA neuronal activity has been reduced after chronic morphine withdrawal. However, this effect has been reversed by restraint stress, which increased the DA release in the NAc core. 3) Stress seems to change the GABA content in both the VTA and NAc regions. GABA levels have decreased in NAc and increased in VTA following the stress, which is reversed by addiction. 4) ECS plays a similar role to addiction, depending on the type of stress, but they have an inhibitory effect on the HPA axis. 5) Following stress, an increase in the content of glutamate and its receptors (GluR1 and AMPARs) in the NAc, as well as an increase in the VTA DA level, can lead to drug cravings. 6) The reduction in 5-HT levels in reward circuits has been seen in stress conditions, while it has increased in addiction. 7) Chronic stress has increased the NE, which has a role in stress-related drug reinstatement. 8) CRF and orexin increments in stressful conditions increased drug-seeking behavior by influencing PFC to regulate addiction-like behaviors. 9) Endogenous BDNF elevation in the NAc subsequently reduced cocaine self-administration and attenuated relapse. 10) The anti-inflammatory cytokine IL-10 expression in the NAc area attenuated the morphine-induced glial activation and prevented the morphine CPP relapse.

The interaction of the HPA axis with other factors or neurotransmitters may open up new avenues for neuroscientists or neuropsychiatrists to discover therapeutics that can be used in addicted subjects. Furthermore, we suggest that more investigations are required to clarify the exact mechanism of stress and its interaction with each factor to affect addiction.

## Ethics approval

All procedures performed in studies were in accordance with the ethical standards of the ethical committee of Kerman University of Medical Sciences (Ethical approval number: IR. KMU.REC.1397.610, Reg. No. 97001069).

## Production notes

### Author contribution statement

All authors listed have significantly contributed to the development and the writing of this article.

### Data availability statement

Data will be made available on request.

### Declaration of interest's statement

The authors declare no conflict of interest.
